# The Association between Statin Use and Reduced Migraine Likelihood: A Comprehensive Analysis of Migraine Subtypes and Statin Types in a Nationwide Korean Cohort

**DOI:** 10.3390/ph17081056

**Published:** 2024-08-10

**Authors:** Ho Suk Kang, Joo-Hee Kim, Ji Hee Kim, Woo Jin Bang, Dae Myoung Yoo, Na-Eun Lee, Kyeong Min Han, Nan Young Kim, Hyo Geun Choi, Kyueng-Whan Min, Mi Jung Kwon

**Affiliations:** 1 Division of Gastroenterology, Department of Internal Medicine, Hallym University Sacred Heart Hospital, Hallym University College of Medicine, Anyang 14068, Republic of Korea; hskang76@hallym.or.kr; 2 Division of Pulmonary, Allergy, and Critical Care Medicine, Department of Medicine, Hallym University Sacred Heart Hospital, Hallym University College of Medicine, Anyang 14068, Republic of Korea; luxjhee@gmail.com; 3 Department of Neurosurgery, Hallym University Sacred Heart Hospital, Hallym University College of Medicine, Anyang 14068, Republic of Korea; kimjihee.ns@gmail.com; 4 Department of Urology, Hallym University Sacred Heart Hospital, Hallym University College of Medicine, Anyang 14068, Republic of Korea; yybbang@hallym.or.kr; 5 Hallym Data Science Laboratory, Hallym University College of Medicine, Anyang 14068, Republic of Korea; ydm@hallym.ac.kr (D.M.Y.); d23009@hallym.ac.kr (N.-E.L.); km.han@hallym.ac.kr (K.M.H.); 6 Laboratory of Brain and Cognitive Sciences for Convergence Medicine, Hallym University College of Medicine, Anyang 14068, Republic of Korea; 7 Hallym Institute of Translational Genomics and Bioinformatics, Hallym University Medical Center, Anyang 14068, Republic of Korea; honeyny@hallym.or.kr; 8 Suseo Seoul E.N.T. Clinic, 10, Bamgogae-ro 1-gil, Gangnam-gu, Seoul 06349, Republic of Korea; mdanalytics@naver.com; 9 Department of Pathology, Uijeongbu Eulji Medical Center, Eulji University School of Medicine, 712, Dongil-ro, Uijeongbu-si 11496, Republic of Korea; kyueng@gmail.com; 10 Department of Pathology, Hallym University Sacred Heart Hospital, Hallym University College of Medicine, Anyang 14068, Republic of Korea

**Keywords:** lipid-lowering agent, statins, lipophilic statin, hydrophilic statin, migraine, nested case–control study

## Abstract

Despite growing interest in the preventive effects of statins, as lipid-lowering agents, on migraine attacks, comprehensive nationwide studies comparing migraine likelihood between statin users and controls are lacking. Our nested case–control study within the Korean National Health Insurance Service-Health Screening Cohort (2002–2019) investigated this association using 38,957 migraine patients and 155,828 controls, considering migraine subtypes (with/without aura) and statin types (lipophilic vs. hydrophilic). Using propensity score matching and adjusting for confounders, statin use was linked to reduced migraine likelihood overall (odds ratio (OR) 0.93), particularly for migraines with aura (OR 0.75) and without aura (OR 0.94). Lipophilic statins were effective for both subtypes, while hydrophilic statins mainly reduced the likelihood of migraines without aura. Subgroup analyses showed consistent benefits across demographics, but varied effectiveness based on weight, smoking, alcohol use, hemoglobin levels, and dyslipidemia history. In summary, this nationwide cohort study suggests that statin use may reduce migraine likelihood among Korean adults across diverse demographics and clinical profiles, but varied effectiveness based on certain lifestyle and comorbidity factors underscores the importance of considering individual patient profiles when assessing the potential benefits of statin therapy for migraine prevention.

## 1. Introduction

Migraine is a complex neurological illness characterized by severe, pulsating headaches, typically affecting one side of the head [1]. It predominantly affects individuals aged 20 to 50 and is recognized as the second most debilitating condition for adults [2]. The prevalence of migraines varies globally, with estimates suggesting that 10–15% of adults experience migraines annually in Europe and America [3]. However, neurologists have reported even higher rates, ranging from 27.6% to 48.6% [4]. Furthermore, approximately 2.5% of those with episodic migraines progress to chronic migraines, which impacts 1–2% of the global population [1]. About one third of all migraine sufferers experience aura—a transient set of neurological symptoms preceding or during attacks, such as visual disturbances or sensory changes caused by changes in brain activity [1]. The presence of aura is linked to an increased risk of stroke and potential connections to cardiovascular disease [5,6], This emphasizes the importance of distinguishing between migraines with and without aura in clinical settings, as the presence of aura may require different management and treatment strategies [7,8].

Various treatment options exist; however, they can cause undesirable side effects [9]. Consequently, alternative treatments with minimal side effects are still under investigation [10]. The accumulating epidemiological [11], experimental [12], meta-analysis [13], and Mendelian randomization [14] studies have identified intriguing associations between plasma lipids and migraines, implying that lipid-lowering agents such as statins could be effective for migraines [15]. Indeed, a case report from about 20 years ago was the first to report that statins completely resolved migraine attacks and prevented recurrence in a migraine patient with hypercholesterolemia [16]. Statins are the most widely prescribed cholesterol-lowering drugs and work by inhibiting the enzyme 3-hydroxy-3-methylglutaryl coenzyme A (HMG-CoA) reductase, which is responsible for the initial step in cholesterol biosynthesis in the liver and brain [17]. Statins are amphiphilic, possessing both hydrophilic and lipophilic regions [18]. Clinically, statins are classified as either hydrophilic or lipophilic based on their balance of polar and non-polar substituents [18]. This classification affects their lipid solubility, bioavailability, and ability to cross the blood–brain barrier; lipophilic statins can cross the barrier due to their high membrane permeability, potentially affecting central nervous system tissues, while hydrophilic statins generally do not and require indirect pathways [18]. Beyond lowering lipids, statins also exhibit anti-inflammatory, antioxidative, immunomodulatory, and vasomotor regulatory properties [17,19], which may have potential benefits for migraine treatment [9].

Despite some studies indicating that statins may reduce migraine risk [20,21,22,23,24], the evidence remains inconclusive, with some reports suggesting that statins might aggravate migraines [25] or induce headaches [26,27]. Most research on this topic has focused on Western populations [15,16,20,21,22,25], with limited studies in other regions [23,24,28], and the existing studies often lack demographic matching and confounder adjustments [20], limiting their generalizability. Moreover, the consistency of associations across diverse patient profiles, including demographic and clinical characteristics, has not been thoroughly explored, suggesting a need for further validation studies. Given the widespread use of statins globally [29], it is crucial to clarify their potential risks or benefits concerning migraines, one of the considerable global burdens [1].

To address these gaps, we undertook a nationwide nested case–control study in Korea, investigating the association between statin use and migraine incidence. Using an established Korean healthcare database, we adjusted for potential confounders and compared the incidence of migraines in statin users to a matched control group. This study advances previous research by conducting detailed subgroup analyses based on sociodemographic and clinical factors, different migraine subtypes, and statin types, and utilizes a precisely matched nationwide dataset.

## 2. Results

### 2.1. Baseline Characteristics

The study included 38,957 migraine patients matched with 155,828 control participants using propensity scores. After matching, both groups had identical demographic characteristics (standardized difference = 0.0) and were well balanced in terms of socioeconomic and lifestyle factors, as well as medical baseline characteristics (standardized difference ≤ 0.2) (Table 1).

### 2.2. Association of Statin Use with Migraine Likelihood

We explored the relationship between statin use and the likelihood of overall migraines and their subtypes (Table 2). The study found that statin use was associated with a decreased likelihood of developing migraines, both with and without aura. The odds ratios (ORs) for overall migraines, migraines with aura, and migraines without aura were 0.93 (95% confidence interval (CI) = 0.91–0.95), 0.75 (95% CI = 0.65–0.86), and 0.94 (95% CI = 0.92–0.96), respectively (all *p* < 0.001).

We further examined the impact of statin type (lipophilic vs. hydrophilic) on the OR of migraine occurrence. Lipophilic statins were associated with reduced incidence of overall migraines (OR = 0.94), migraines with aura (OR = 0.75), and migraines without aura (OR = 0.95) (all *p* < 0.001) (Table 3).

Hydrophilic statins were linked to a reduced likelihood of overall migraines (OR = 0.92) and migraines without aura (OR = 0.93) (all *p* < 0.001), but the association with migraines with aura was not statistically significant (OR = 0.71, *p* = 0.080) (Table 4).

### 2.3. Subgroup Analyses 

**Figure 1:**
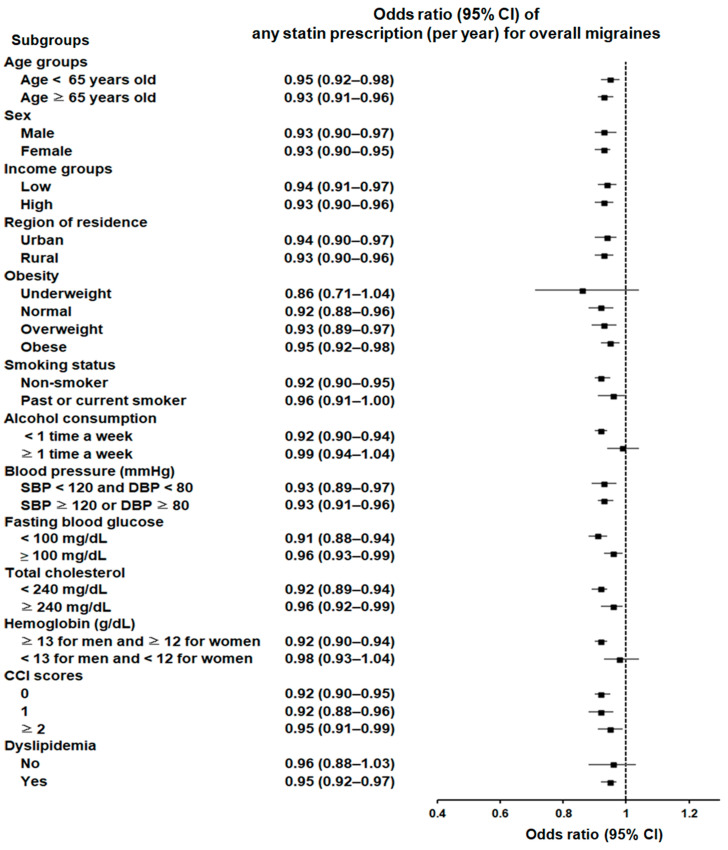
Forest plot depicting odds ratios (95% confidence intervals [CIs]) of incident migraines according to any statin type.

Subgroup analyses showed a consistent significant association between statin use and reduced likelihood of migraines across various demographic and clinical characteristics. Statin use was linked to a lower probability of migraines across all age groups and both sexes, regardless of income status, residential region, blood pressure, fasting blood glucose level, total cholesterol level, or Charlson Comorbidity Index (CCI) score. This association was also maintained among individuals who were of normal weight, overweight, or obese; non-smokers; those who engage in infrequent alcohol consumption; individuals without anemia; and those with a history of dyslipidemia (Figure 1; Supplementary Table S1).

**Figure 2:**
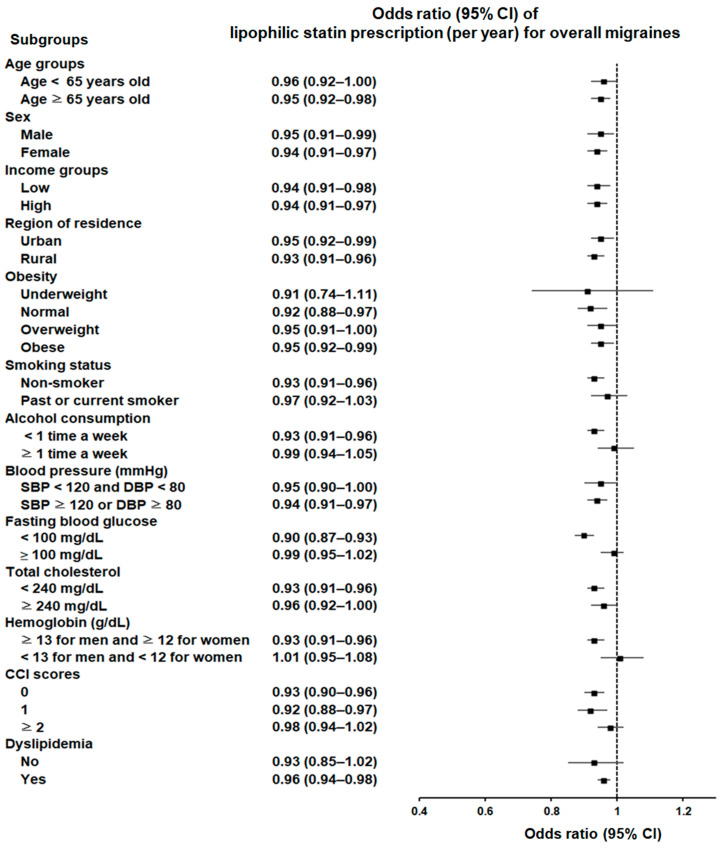
Forest plot depicting odds ratios (95% confidence intervals [CIs]) of incident migraines according to lipophilic statins.

In terms of statin types, lipophilic statin use was associated with a decreased likelihood of migraines across diverse subgroups, including various age groups; both sexes; and assorted income statuses, residential regions, blood pressure levels, and CCI scores. This association remained significant among individuals who were of normal weight, overweight, or obese; non-smokers; those who engage in infrequent alcohol consumption; individuals without anemia; those with a history of dyslipidemia; and those with specific fasting blood glucose levels (<100 mg/dL) (Figure 2; Supplementary Table S2).

**Figure 3:**
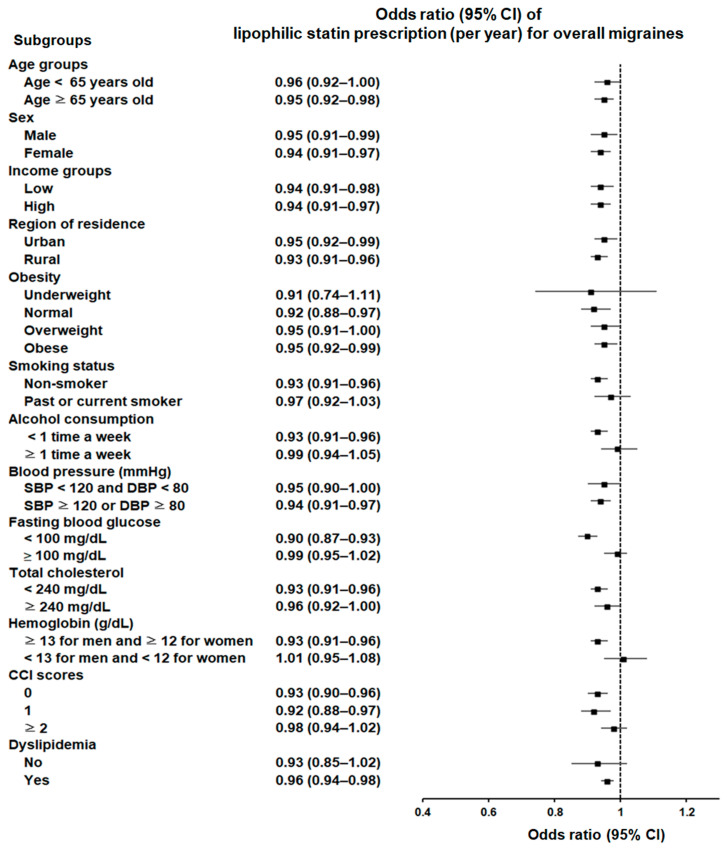
Forest plot depicting odds ratios (95% confidence intervals [CIs]) of incident migraines according to hydrophilic statins.

Hydrophilic statin use also showed a significant association with a reduced probability of migraines, independent of sex, income status, residential area, blood pressure, or CCI score. This association was significant among individuals aged 65 years and older; those with an overweight status; non-smokers; those who engage in infrequent alcohol consumption; those without anemia; those with specific levels of fasting blood glucose (≥100 mg/dL); and those with a history of dyslipidemia (Figure 3; Supplementary Table S3).

## 3. Discussion

Despite growing interest in the effect of statins on migraine reduction [23,24,28], large-scale nationwide population-based studies comparing the incidence of migraines in statin users to matched control groups are still lacking. Our well-balanced, large-scale nationwide nested case–control study revealed a consistent and significant decline in the odds of developing migraines among Korean adults using statins. Multivariable logistic regression analysis—adjusted for demographics, socioeconomics, lifestyle, and comorbidities—indicated that statin use may independently reduce migraine probability by 5% to 25%, depending on the migraine type, statin type, and population subgroup. Both lipophilic and hydrophilic statins demonstrated protective effects, though with some differences in efficacy depending on the migraine subtype. Lipophilic statins significantly reduced the likelihood of both migraines with aura and migraines without aura, while hydrophilic statins were primarily effective in reducing migraines without aura. Subgroup analyses showed that the protective effect of overall statin use was consistent across various demographics and clinical profiles, regardless of sex, age, income, region, CCI score, fasting blood glucose, blood pressure, or total cholesterol. These findings suggest the potential efficacy of statins in managing migraines across diverse patient populations, indicating a broad potential for reducing migraine occurrence with statin use.

Our findings support previous research on the potential protective role of statins in migraine patients. Since the first case report in 2006 of using statins for successful migraine treatment [16], most subsequent observational studies have indicated a beneficial effect of statins on migraines [20,22]. Three recent small-scale clinical trials have demonstrated that statins could be an effective and safe alternative for other medications in migraine prophylaxis, with comparable efficacy and fewer adverse effects [23,24,28]. An earlier open-label and preliminary prospective study showed a significant reduction in migraine attacks with statin treatment over three months, with 83% of patients experiencing more than a 50% reduction in migraine frequency [22]. However, the study’s small sample size (*n* = 29) and inclusion of only female patients [22] may introduce selection bias and limit the generalizability of the results. Nevertheless, this study paved the way for further epidemiologic and randomized controlled studies, as well as animal models, to evaluate whether statins prevent migraine. A subsequent cross-sectional population-based study including 5938 US individuals aged ≥40 years demonstrated that the use of statins was linked to a 33% lower risk of experiencing severe headaches or migraines (95% CI = 0.46–0.98) [20]. However, this study relied on self-reported health and medication status [20], making it vulnerable to recall bias, and did not differentiate between migraines and headaches diagnosed by health professionals. The same authors later performed a randomized controlled trial demonstrating that the combination of simvastatin and vitamin D effectively prevents headaches in adults with episodic migraine, with strict eligibility criteria [21]. On the other hand, a few publications suggest that statin use might be connected to an elevated risk of migraines [25] or headaches [26,27]. However, these associations appear weak and are often based on case reports under extreme environmental conditions, such as high altitudes [25]. Some people experience headaches, not specifically migraines, while taking certain statins, including pravastatin [26] or rosuvastatin [27]. To prevent selection bias and heterogeneity, we utilized a methodologically rigorous study design with nationwide population-based controls, thoroughly considering all possible confounders. Our study included only patients who had more than two clinic visits for migraines diagnosed by neurology specialists, and excluded those with other types of headaches diagnosed at baseline. We also applied a three-year washout period to avoid enrolling pre-existing migraine cases in the study. This rigorous approach aimed to increase the validity of our study, providing more accurate and reliable findings regarding the relationship between statin use and the probability of migraines.

Additionally, our findings extend previous research by examining the association of statin use with migraine likelihood through subgroup analyses of diverse clinical profiles, migraine subtypes, and statin lipophilicity types. Unlike previous cohort studies that did not consider these subgroup differences [20,22], our subgroup analyses revealed a consistent link between overall statin use and decreased odds of migraine, independent of sex, income status, residential area, blood pressure, fasting blood glucose, total cholesterol, or CCI score. This may suggest broad preventive implications for statin use in migraine management across diverse patient demographics and comorbid conditions. Considering that migraine sufferers often have a higher cardiovascular risk profile and elevated cholesterol levels [30], the potential clinical benefits of statins in preventing migraines for these high-risk cardiometabolic individuals are clinically noteworthy.

However, variations in effectiveness were observed with certain statin lipophilicity types among subgroups, including different weight statuses, smoking habits, alcohol consumption patterns, hemoglobin levels, and dyslipidemia history. The reductive effects of both lipophilic and hydrophilic statins on the likelihood of migraines remained significant among participants in the present study who were overweight, did not smoke, consumed alcohol infrequently, were not anemic, and had a history of dyslipidemia. Since the acknowledged risk factors of migraine include alcohol consumption, smoking, obesity, and anemia [1,31,32], individuals without these risk factors may benefit more from the use of statins to reduce the likelihood of migraine. Our findings emphasize the importance of evaluating individual patient profiles when assessing the potential benefits of statin therapy for migraine prevention.

The effectiveness of statins in reducing the likelihood of migraine also seems to vary according to different migraine subtypes. Our study revealed that migraines with aura benefited the most from statin use, with a 25% reduced likelihood when using any statins or lipophilic statins. In contrast, migraines without aura and overall migraines showed smaller reductions (5–7%). Lipophilic statins significantly reduced the likelihood of migraines both with and without aura, while hydrophilic statins primarily reduced migraines without aura in our study. Limited data exist on how different statin lipophilicity types can affect migraine subtypes, but case reports and studies suggest that lipophilic statins often seem to be more effective [12,16,21,22,23,24] than hydrophilic statins [28]. For instance, atorvastatin (a lipophilic type) effectively resolved the issue of migraines with aura in a patient with dyslipidemia and peripheral arterial disease [16]. Two clinical trials in Iran confirmed that atorvastatin significantly reduced migraine attacks with fewer adverse effects [23,24], achieving a 65% response rate for migraines with aura [24] compared to the 13% response rate of conventional therapy [33]. Conversely, another trial revealed that rosuvastatin (a hydrophilic type) combined with propranolol reduced migraine attacks, but the subtypes were not specified [28]. Migraines with aura involve decreased cerebral blood flow and cortical depolarization, causing peptide release, inflammation, and vessel dilation [34]. Statins, which are detectable in the brain after a single dose, inhibit the production of crucial compounds such as cholesterol, coenzyme Q, prenylated proteins, and dolichol, which are essential for brain function and development [35]. Lipophilic statins (e.g., lovastatin and simvastatin) cross the blood–brain barrier directly [12,36], while hydrophilic statins have weak lipophilic properties, making it difficult for them to cross the blood–brain barrier [35,36]. Consequently, they rely on active transporters to enter the brain indirectly [35,36]. Compared to hydrophilic statins, lipophilic statins are more likely to provide superior vascular anti-inflammatory effects and immune suppression [17,36], potentially reducing the risk of developing migraines with aura more effectively.

The mechanisms behind the association between reduced migraine likelihood and statin use are complex and not fully understood. Statins may improve migraine pathophysiology through neuroprotective actions and genetic susceptibility. Migraine pain originates from disrupted neural networks governed by the brainstem and diencephalic nuclei, which regulate the trigeminovascular system [9]. This system involves efferent neurons that supply vascular networks and afferent neurons that transmit information to the trigeminal nucleus caudalis [10]. Activation of NOD-like receptor protein 3 (NLRP3) in this region contributes to inflammation of the microglial–neuronal signaling pathways, leading to meningeal inflammation and vasodilation [9,19]. This inflammation leads to elevated levels of C-reactive proteins, interleukins (such as IL-1 and IL-6), tumor necrosis factor-alpha (TNF-α), and adhesion molecules [37], all of which indicate systemic inflammation, oxidative stress, and thrombosis [9]. By inhibiting HMG-CoA reductase, statins downregulate protein prenylation, suppress NADPH oxidase activity, and reduce pro-inflammatory cytokines such as IL-1β, IL-6, IL-8, and TNF-α in the brain [35,37]. This modulation of neuronal processes, synaptic transmission, neuroinflammation, and oxidative stress may specifically target the trigeminal nucleus caudalis [35], which is involved in migraines. In animal models, statins attenuate the activation of the nuclear factor-kB signaling pathway, which is responsible for neuronal plasticity in this region, potentially inhibiting pain transmission to the cortex during migraine attacks [12]. Overall, these actions may contribute to decreased activation of the disrupted neural networks associated with migraines. Interestingly, a recent Mendelian randomization study using genome-wide association data from a population of European descent showed that high expression of the *HMGCR* gene, which encodes HMG-CoA reductase, correlates with a 1.55 times higher risk of migraines (95% CI 1.30–1.84) [15]. Another study showed that single-nucleotide polymorphisms related to HMG-CoA reductase are linked to an increased risk of overall migraines and migraines with aura [38]. Additionally, a separate Mendelian randomization study revealed that genetic indicators for HMG-CoA reductase inhibition are linked to a 36% reduced likelihood of migraines in the FinnGen dataset (95% CI 0.46–0.88) [14]. These findings support the notion that HMG-CoA reductase inhibitors, or statins, may lower susceptibility to migraines.

Metabolic abnormalities such as oxidative stress in peripheral tissues, issues with glucose metabolism, and dysfunction of mitochondrial enzymes are linked to migraines [8,39], which may be alleviated by statins. An animal study demonstrated that a combination of atorvastatin and fluvastatin significantly improved metabolic, mitochondrial, and physical dysfunctions in rats subjected to chronic running wheel activity [40]. Additionally, serotonin, a critical neurotransmitter in migraine pathophysiology and treatment, is modulated to enhance the serotonin signal, providing pain relief through vasoconstriction and peptide inhibition [41]. Simvastatin has been shown to counteract the upregulation of the serotonergic pathway by inhibiting indoleamine 2,3-dioxygenase, thereby increasing tryptophan and serotonin levels [42].

This study’s strengths stem from its use of extensive, representative, nationwide population-based data. Comprehensive medical histories from every clinic and hospital across Korea enhance the accuracy and generalizability of the findings. To reduce selection bias and enhance precision, two groups (38,957 individuals with migraines and 155,828 individuals without migraines) were matched using propensity scores. This matching ensured equitable adjustments for socioeconomic status and potential risk factors and comorbidities related to migraines or statin use. Additionally, the thorough consideration of possible confounding factors, such as socioeconomic status, comorbidities, and lifestyle-related risk factors, further bolsters the study’s validity. Subgroup analyses further support the consistent protective effect of statins across various demographics and clinical profiles.

However, a few limitations should be considered when reviewing our findings. First, due to the observational and retrospective nature of our study design, we cannot conclusively determine a causal relationship between statin use and migraines, and we also did not explore the underlying mechanisms that could explain the association between statin use and a reduced likelihood of migraines. Second, our study focused exclusively on Korean citizens over 40 years old and utilized diagnosis codes from Korean health insurance data. This may result in some unmeasured confounding variables and restrict the generalizability of our findings in terms of other populations. Third, the Korean National Health Insurance Service-Health Screening Cohort database used in our study lacked information on migraine severity, family history, genetics, and dietary habits, which may have limited our ability to comprehensively understand and investigate the relationship between statin use and the likelihood of migraines. Fourth, we recognize that our analysis categorized statins broadly into hydrophilic and lipophilic groups, without accounting for the specific differences among the individual compounds within these groups. Each statin has unique physicochemical and pharmacological properties that may influence its effectiveness in reducing migraine occurrence. This variability among individual compounds is a limitation of our study. Additionally, we did not assess the impact of the intensity of the prescribed statin dosage, which could also play a significant role in statins’ efficacy. We suggest that future research should explore the effects of specific statins, taking into account both their lipophilicity and the dosage intensity, to gain a more comprehensive understanding of their impact on migraines. Interestingly, genetic variations in other lipid-lowering targets, such as APOB or PCSK9, which are associated with higher LDL cholesterol levels, have also been linked to a reduced risk of migraines [38]. Additionally, LPL inhibitors have shown significant associations with migraine risk [14]. These observations highlight the potential for other lipid-modifying treatments to impact migraine occurrence. For instance, CETP inhibitors, despite not achieving the expected benefit of reducing cardiovascular disease risk, may warrant further investigation for their effects on migraine occurrence [14,38]. Future studies should explore the broader implications of these treatments, in particular, to understand their specific roles and potential benefits in individuals with migraines.

## 4. Materials and Methods

### 4.1. Research Methodology and Subjects 

This study leveraged the Korean National Health Insurance Service-Health Screening Cohort database, a valuable resource for policies and research. The Korean National Health Insurance Service has provided mandatory health insurance to about 97% of the population since 1999. The Korean National Health Insurance Service-Health Screening Cohort consists of anonymized data and the International Classification of Diseases, Tenth Revision, Clinical Modification (ICD-10-CM) diagnostic codes from 514,866 individuals aged 40–79 who had health screenings in 2002–2003, with follow-up data until 2019 [43]. Participants were chosen through a 10% simple random sampling method [43]. The study was ethically approved (approval no. 2019-10-023), and a waiver for written informed consent was granted due to the use of secondary data.

**Figure 4:**
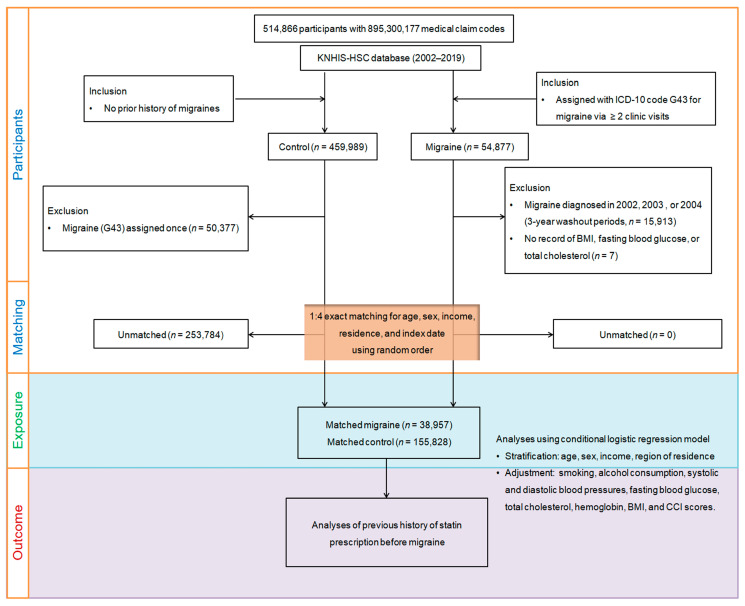
A schematic depiction of the participant selection method employed in this study. From the original pool of 514,866 participants in the Korean National Health Insurance Service-Health Screening Cohort (KNHIS-HSC) database, a detailed matching process paired 38,957 migraine sufferers with 155,828 control participants, considering propensity scores. ICD-10, International Classification of Diseases, Tenth Revision; BMI, body mass index; CCI, Charlson Comorbidity Index.

We initially identified 54,877 patients newly diagnosed with migraine between 2002 and 2019, using data from the extensive Korean National Health Insurance Service-Health Screening Cohort database, which includes 514,866 adult patients (aged 40 and above) and 895,300,177 medical claim codes (Figure 4). The index date for each migraine patient was the day the ICD-10 code for migraine (G43) was first recorded in their health insurance claims. To minimize false positives, we included only patients with more than two clinic visits for migraines diagnosed with the ICD-10 code G43. We excluded 15,913 patients diagnosed with migraine in 2002, 2003, and 2004 to avoid including pre-existing cases, and 7 patients with no health screening records. On the other hand, the initial control group comprised 459,989 participants without a migraine diagnosis from 2002 to 2019. We excluded 50,377 participants who had been diagnosed with migraine even once during this period.

Propensity score matching was used to pair each migraine patient with a control participant based on region, income, sex, and age, ensuring optimal balance between the two groups, along with random clustered sorting. Each migraine patient was matched with a control participant sharing the same index date. This process resulted in the elimination of 253,784 control members, leaving no unmatched migraine patients. Ultimately, we successfully matched 38,957 migraine patients to 155,828 controls at a 1:4 ratio. 

### 4.2. Outcomes (Migraine)

The study enrolled migraine patients who had at least two treatments for ICD-10 code G43, given by neurology specialists, between 2002 and 2019, excluding those diagnosed with other headaches (ICD-10 code G44) [6,44]. Patients were classified as having migraines with aura if diagnosed with or treated for ICD-10 code G431 (migraine with aura), and were otherwise classified as having migraines without aura. The primary outcome was the likelihood of developing migraines with statin use (any type, lipophilic, or hydrophilic), and the secondary outcome examined the likelihood of migraines with or without aura in relation to statin use.

### 4.3. Exposure (Statin)

We retrospectively identified statin prescriptions prior to migraine diagnosis over a 2-year period before the index dates, classifying patients as statin users if they had prescription history within this timeframe [45]. The prescribed statins included simvastatin, atorvastatin, pravastatin, lovastatin, rosuvastatin, and fluvastatin. These were categorized into lipophilic (fluvastatin, lovastatin, simvastatin, atorvastatin) and hydrophilic statins (rosuvastatin, pravastatin) to assess the impact of lipid affinity on migraine occurrence. The average drug use per year was calculated to evaluate statin exposure.

### 4.4. Covariates

Participants were divided into ten age groups (40–44 years to 85+ years), five income groups (Class 1 [lowest income] to Class 5 [highest income]), and two regional groups (urban and rural). They were also classified by smoking status, alcohol consumption, and BMI-based weight status. Data on total cholesterol, blood pressure, fasting blood glucose, and hemoglobin were collected. The CCI was used to assess comorbidity severity, with scores ranging from 0 to 29 [46]. Dyslipidemia (ICD-10 code: E78) was identified if participants had received treatment at least twice.

### 4.5. Statistical Analyses

To address confounding factors and selection bias, we used propensity score matching [47], calculated via multivariable logistic regression with baseline covariates, and paired migraine patients with controls using a nearest-neighbor algorithm [48]. We ensured balance by inspecting absolute standardized differences (≤0.20 indicated good balance) [47,48]. Additional adjustments were made with multivariable logistic regression for covariates exceeding 0.20 [47]. Data are presented as numbers and percentages for categorical variables, and as means with standard deviations for continuous variables.

The relationship between statin prescriptions (per year) and migraine outcomes was analyzed by using conditional logistic regression models to determine ORs and 95% CIs. Three models were used: a crude model (controlling for age, sex, income, and residence); an adjusted model (Model 1) for potential confounders (blood pressure, fasting blood glucose, total cholesterol, hemoglobin, and dyslipidemia history); and a further adjusted model (Model 2) for additional factors (obesity status, smoking, alcohol consumption, statin type, and CCI score). The effects of hydrophilic and lipophilic statins were analyzed separately and together, with subgroup analyses including all covariates for overall migraines. Statistical analysis was performed using SAS version 9.4, with *p*-values lower than 0.05 considered significant.

## 5. Conclusions

In summary, this extensive cohort study may add supporting evidence for the beneficial impact of statins on migraine occurrence. The results indicate that statin use may lower the likelihood of migraines developing in Korean adults, irrespective of sex, age, income, region, blood pressure, fasting blood glucose, total cholesterol, or CCI score, but effectiveness varies based on weight, smoking, alcohol use, hemoglobin levels, and dyslipidemia history, underscoring the importance of considering individual patient profiles when assessing the potential benefits of statin therapy for migraine prevention. Both lipophilic and hydrophilic statins may show protective effects against migraines, with lipophilic statins being particularly effective for migraines with aura. Understanding the differential effects of statin lipophilicity types on migraine subtypes might lead to more precise and efficacious treatments for migraine sufferers.

## Figures and Tables

**Table 1:** General characteristics of participants.

Characteristics	Total Participants		
	Migraine (n, %)	Control (n, %)	Standardized Difference
Total number	38,957 (100.0)	155,828 (100.0)	
Age (years old)			0.00
40–44	449 (1.15)	1796 (1.15)	
45–49	3736 (9.59)	14,944 (9.59)	
50–54	6092 (15.64)	24,368 (15.64)	
55–59	6665 (17.11)	26,660 (17.11)	
60–64	5988 (15.37)	23,952 (15.37)	
65–69	6048 (15.52)	24,192 (15.52)	
70–74	5024 (12.90)	20,096 (12.90)	
75–79	3129 (8.03)	12,516 (8.03)	
80–84	1399 (3.59)	5596 (3.59)	
85+	427 (1.10)	1708 (1.10)	
Sex			0.00
Male	13,494 (34.64)	53,976 (34.64)	
Female	25,463 (65.36)	101,852 (65.36)	
Income			0.00
1 (lowest)	7284 (18.70)	29,136 (18.70)	
2	5435 (13.95)	21,740 (13.95)	
3	6329 (16.25)	25,316 (16.25)	
4	8107 (20.81)	32,428 (20.81)	
5 (highest)	11,802 (30.29)	47,208 (30.29)	
Region of residence			0.00
Urban	15,307 (39.29)	61,228 (39.29)	
Rural	23,650 (60.71)	94,600 (60.71)	
Weight status †			0.07
Underweight	935 (2.40)	3922 (2.52)	
Normal	13,868 (35.60)	55,818 (35.82)	
Overweight	10,543 (27.06)	41,788 (26.82)	
Obese I	12,379 (31.78)	48,938 (31.41)	
Obese II	1232 (3.16)	5362 (3.44)	
Smoking status			0.03
Non-smoker	30,883 (79.27)	121,898 (78.23)	
Past smoker	2297 (5.90)	9440 (6.06)	
Current smoker	5777 (14.83)	24,490 (15.72)	
Alcohol consumption			0.03
<1 time a week	31,602 (81.12)	124,345 (79.80)	
≥1 time a week	7355 (18.88)	31,483 (20.20)	
Systolic blood pressure (mean, SD) (mmHg)	125.66 (16.25)	126.71 (17.03)	0.06
Diastolic blood pressure (mean, SD) (mmHg)	77.40 (10.41)	77.83 (10.67)	0.04
Fasting blood glucose (mean, SD) (mg/dL)	98.96 (25.87)	100.85 (29.26)	0.07
Total cholesterol (mean, SD) (mg/dL)	199.61 (38.44)	199.61 (38.51)	0.00
Hemoglobin (mean, SD) (g/dL)	13.48 (1.43)	13.49 (1.46)	0.01
CCI score (mean, SD)	1.02 (1.61)	0.95 (1.67)	0.04
Migraines with/without aura			
Migraines with aura (n, %)	3643 (9.35)	0 (0.0)	
Migraines without aura (n, %)	35,314 (90.65)	0 (0.0)	

Abbreviations: CCI, Charlson Comorbidity Index; SD, standard deviation. † Obesity (BMI, body mass index, kg/m^2^) was categorized as <18.5 (underweight), ≥18.5 to <23 (normal), ≥23 to <25 (overweight), ≥25 to <30 (obese I), and ≥30 (obese II).

**Table 2:** Crude and adjusted odds ratios (95% confidence interval) of statin prescription (per 1 year) for overall migraines, migraines with aura, and migraines without aura.

Characteristics	OR (95% CI)					
	Crude †	*p*	Model 1 †‡	*p*	Model 2 †§	*p*
OR of statins for overall migraines	1.04 (1.02–1.06)	<0.001 *	0.93 (0.91–0.95)	<0.001 *	0.93 (0.91–0.95)	<0.001 *
OR of statins for migraines with aura	0.85 (0.74–0.98)	0.023 *	0.75 (0.65–0.87)	<0.001 *	0.75 (0.65–0.86)	<0.001 *
OR of statins for migraines without aura	1.04 (1.02–1.07)	<0.001 *	0.94 (0.91–0.96)	<0.001 *	0.94 (0.92–0.96)	<0.001 *

Abbreviations: OR, odds ratio; 95% CI, 95% confidence interval. * Conditional logistic regression model, significant at *p* < 0.05. † Models were stratified by age, sex, income, and region of residence. ‡ Model 1 was adjusted for systolic blood pressure, diastolic blood pressure, fasting blood glucose, total cholesterol, and hemoglobin level. § Model 2 was adjusted for Model 1 plus obesity, smoking, alcohol consumption, and Charlson Comorbidity Index (CCI) score.

**Table 3:** Crude and adjusted odds ratios (95% confidence interval) of lipophilic statin prescription (per 1 year) for overall migraines, migraines with aura, and migraines without aura.

Characteristics	OR (95% CI)					
	Crude †	*p*	Model 1 †‡	*p*	Model 2 †§	*p*
OR of lipophilic statins for overall migraines	1.04 (1.02–1.07)	<0.001 *	0.94 (0.92–0.96)	<0.001 *	0.94 (0.92–0.97)	<0.001 *
OR of lipophilic statins for migraines with aura	0.86 (0.74–1.00)	0.046 *	0.76 (0.65–0.88)	<0.001 *	0.75 (0.64–0.88)	<0.001 *
OR of lipophilic statins for migraines without aura	1.05 (1.03–1.07)	<0.001 *	0.95 (0.92–0.97)	<0.001 *	0.95 (0.93–0.97)	<0.001 *

Abbreviations: OR, odds ratio; 95% CI, 95% confidence interval. * Conditional logistic regression model, significant at *p* < 0.05. † Models were stratified by age, sex, income, and region of residence. ‡ Model 1 was adjusted for systolic blood pressure, diastolic blood pressure, fasting blood glucose, total cholesterol, and hemoglobin level. § Model 2 was adjusted for Model 1 plus obesity, smoking, alcohol consumption, and Charlson Comorbidity Index (CCI) score.

**Table 4:** Crude and adjusted odds ratios (95% confidence interval) of hydrophilic statin prescription (per 1 year) for overall migraines, migraines with aura, and migraines without aura.

Characteristics	OR (95% CI)					
	Crude †	*p*	Model 1 †‡	*p*	Model 2 †§	*p*
OR of hydrophilic statins for overall migraines	1.01 (0.97–1.05)	0.579	0.92 (0.88–0.96)	<0.001 *	0.92 (0.88–0.96)	<0.001 *
OR of hydrophilic statins for migraines with aura	0.80 (0.55–1.17)	0.254	0.71 (0.49–1.04)	0.077	0.71 (0.49–1.04)	0.080
OR of hydrophilic statins for migraines without aura	1.01 (0.97–1.06)	0.485	0.92 (0.88–0.96)	<0.001 *	0.93 (0.89–0.96)	<0.001 *

Abbreviations: OR, odds ratio; 95% CI, 95% confidence interval. * Conditional logistic regression model, significant at *p* < 0.05. † Models were stratified by age, sex, income, and region of residence. ‡ Model 1 was adjusted for systolic blood pressure, diastolic blood pressure, fasting blood glucose, total cholesterol, and hemoglobin level. § Model 2 was adjusted for Model 1 plus obesity, smoking, alcohol consumption, and Charlson Comorbidity Index (CCI) score.

## Data Availability

All data are available from the National Health Insurance Sharing Service (NHISS) database (https://nhiss.nhis.or.kr; accessed on 1 February 2024). The NHISS allows access to all these data for any researcher who promises to follow the research ethics guidelines and pays a processing charge. If you wish to access the data used in this article, you can download them from the website after promising to follow the research ethics.

## Supplementary Materials

The following supporting information can be downloaded at: https://www.mdpi.com/article/10.3390/ph17081056/s1. Supplementary Table S1: Subgroup analyses of crude and adjusted odds ratios (95% confidence interval) of statin prescription (per 1 year) for overall migraines. Supplementary Table S2: Subgroup analyses of crude and adjusted odds ratios (95% confidence interval) of lipophilic statin prescription (per 1 year) for overall migraines. Supplementary Table S3: Subgroup analyses of crude and adjusted odds ratios (95% confidence interval) of hydrophilic statin prescription (per 1 year) for overall migraines.
