# Supplementary material for: The Association between Statin Use and Reduced Migraine Likelihood: A Comprehensive Analysis of Migraine Subtypes and Statin Types in a Nationwide Korean Cohort
Source: Pharmaceuticals (Basel). 2024 Aug 10;17(8):1056. doi: 10.3390/ph17081056 (PMC11357270; doi:10.3390/ph17081056)
Supplement: Supplementary file 1 [file pharmaceuticals-17-01056-s001.zip › pharmaceuticals-3135448-supplementary.pdf]

Table S1 Subgroup analyses of crude and adjusted odds ratios (95% confidence interval) of statin prescription (per 1 year) for overall migraines

Characteristics	Odds ratios (95% CI)					
	Crude†	P-value	Model 1†‡	P-value	Model 2†§	P-value
**Age < 65 years old (n =114,650)**						
Statin prescription	1.08 (1.04-1.11)	<0.001*	0.95 (0.92-0.99)	0.005*	0.95 (0.92-0.98)	0.005*
**Age ≥ 65 years old (n =80,135)**						
Statin prescription	1.02 (0.99-1.04)	0.246	0.93 (0.90-0.96)	<0.001*	0.93 (0.91-0.96)	<0.001*
**Men (n =67,470)**						
Statin prescription	1.05 (1.02-1.09)	0.003*	0.93 (0.90-0.97)	<0.001*	0.93 (0.90-0.97)	<0.001*
**Women (n =95,240)**						
Statin prescription	1.03 (1.00-1.06)	0.021*	0.93 (0.90-0.95)	<0.001*	0.93 (0.90-0.95)	<0.001*
**Low income (n =95,240)**						
Statin prescription	1.05 (1.02-1.09)	<0.001*	0.94 (0.91-0.97)	<0.001*	0.94 (0.91-0.97)	<0.001*
**High income (n =99,545)**						
Statin prescription	1.03 (1.00-1.05)	0.070	0.92 (0.90-0.95)	<0.001*	0.93 (0.90-0.96)	<0.001*
**Urban residents (n =76,535)**						
Statin prescription	1.05 (1.02-1.08)	0.003*	0.93 (0.90-0.96)	<0.001*	0.94 (0.90-0.97)	<0.001*
**Rural residents (n =118,250)**						
Statin prescription	1.03 (1.00-1.06)	0.022*	0.93 (0.90-0.95)	<0.001*	0.93 (0.90-0.96)	<0.001*
**Underweight (n =4,857)**						
Statin prescription	1.06 (0.89-1.26)	0.519	0.86 (0.71-1.04)	0.118	0.86 (0.71-1.04)	0.117
**Normal weight (n =69,686)**						
Statin prescription	1.05 (1.01-1.09)	0.009*	0.92 (0.88-0.96)	<0.001*	0.92 (0.88-0.96)	<0.001*
**Overweight (n =52,331)**						
Statin prescription	1.04 (1.00-1.08)	0.035*	0.92 (0.89-0.96)	<0.001*	0.93 (0.89-0.97)	<0.001*
**Obese (n =67,911)**						
Statin prescription	1.02 (0.99-1.05)	0.166	0.94 (0.91-0.97)	<0.001*	0.95 (0.92-0.98)	0.001*
**Nonsmoker (n =152,781)**						
Statin prescription	1.02 (1.00-1.04)	0.091	0.92 (0.90-0.94)	<0.001*	0.92 (0.90-0.95)	<0.001*
**Past smoker and current smoker (n =42,004)**						
Statin prescription	1.10 (1.05-1.15)	<0.001*	0.96 (0.91-1.00)	0.061	0.96 (0.91-1.00)	0.055

**Alcohol consumption < 1 time a week (n =155,947)**						
Statin prescription	1.02 (1.00-1.04)	0.078	0.92 (0.89-0.94)	<0.001*	0.92 (0.90-0.94)	<0.001*
**Alcohol consumption ≥ 1 time a week (n =38,838)**						
Statin prescription	1.11 (1.06-1.16)	<0.001*	0.98 (0.94-1.03)	0.511	0.99 (0.94-1.04)	0.587
**Systolic blood pressure < 120 mmHg and diastolic blood pressure < 80 mmHg (n =55,815)**						
**Statin prescription**	1.08 (1.04-1.13)	<0.001*	0.93 (0.89-0.97)	0.001*	0.93 (0.89-0.97)	0.001*
**Systolic blood pressure ≥ 120 mmHg or diastolic blood pressure ≥ 80 mmHg (n =138,970)**						
Statin prescription	1.03 (1.00-1.05)	0.025*	0.93 (0.91-0.95)	<0.001*	0.93 (0.91-0.96)	<0.001*
**Fasting blood glucose < 100 mg/dL (n =122,997)**						
Statin prescription	1.05 (1.02-1.08)	0.001*	0.91 (0.88-0.93)	<0.001*	0.91 (0.88-0.94)	<0.001*
**Fasting blood glucose ≥ 100 mg/dL (n =71,788)**						
Statin prescription	1.06 (1.03-1.09)	<0.001*	0.96 (0.93-0.99)	0.005*	0.96 (0.93-0.99)	0.007*
**Total cholesterol < 200 mg/dL (n =102,102)**						
Statin prescription	1.03 (1.01-1.06)	0.011*	0.92 (0.89-0.94)	<0.001*	0.92 (0.89-0.94)	<0.001*
**Total cholesterol ≥ 200 mg/dL (n =92,683)**						
Statin prescription	1.05 (1.01-1.08)	0.012*	0.96 (0.92-0.99)	0.015*	0.96 (0.92-0.99)	0.016*
**Hemoglobin ≥ 13 g/dL for men and ≥ 12 g/dL for women (n =168,718)**						
Statin prescription	1.03 (1.00-1.05)	0.021*	0.92 (0.90-0.94)	<0.001*	0.92 (0.90-0.94)	<0.001*
**Hemoglobin < 13 g/dL for men and < 12 g/dL for women (n =26,067)**						
Statin prescription	1.10 (1.04-1.15)	<0.001*	0.98 (0.93-1.04)	0.584	0.98 (0.93-1.04)	0.592
**CCI scores = 0 (n =117,143)**						
Statin prescription	1.04 (1.01-1.07)	0.003*	0.92 (0.89-0.95)	<0.001*	0.92 (0.90-0.95)	<0.001*
**CCI scores = 1 (n =32,408)**						
Statin prescription	0.96 (0.92-1.00)	0.041*	0.92 (0.87-0.96)	<0.001*	0.92 (0.88-0.96)	<0.001*
**CCI scores ≥ 2 (n =45,234)**						
Statin prescription	1.02 (0.99-1.06)	0.212	0.95 (0.92-0.99)	0.016*	0.95 (0.91-0.99)	<0.001*
**No dyslipidemia history (n =85,094)**						
Statin prescription	0.97 (0.90-1.05)	0.461	0.96 (0.89-1.04)	0.302	0.96 (0.88-1.03)	0.246
**Dyslipidemia history (n =109,691)**						
Statin prescription	0.92 (0.90-0.94)	<0.001*	0.94 (0.92-0.96)	<0.001*	0.95 (0.92-0.97)	<0.001*

Abbreviation: CCI, Charlson Comorbidity Index.* Conditional or unconditional logistic regression model, Significance at P < 0.05.† Models were stratified by age, sex, income, and region of residence. ‡ Model 1 was adjusted for systolic blood pressure, diastolic blood pressure, fasting blood glucose, total cholesterol, and hemoglobin levels.

Table S2 Subgroup analyses of crude and adjusted odds ratios (95% confidence interval) of lipophilic statin prescription (per 1 year) for overall migraines

Characteristics	Odds ratios (95% CI)					
	Crude†	P-value	Model 1†‡	P-value	Model 2†§	P-value
**Age < 65 years old (n = 114,650)**						
Lipophilic statin prescription	1.08 (1.04-1.12)	<0.001*	0.96 (0.92-1.00)	0.038*	0.96 (0.92-1.00)	0.031*
**Age ≥ 65 years old (n = 80,135)**						
Lipophilic statin prescription	1.02 (0.99-1.05)	0.137	0.94 (0.92-0.97)	<0.001*	0.95 (0.92-0.98)	<0.001*
**Men (n = 67,470)**						
Lipophilic statin prescription	1.06 (1.02-1.10)	0.003*	0.94 (0.91-0.98)	0.006*	0.95 (0.91-0.99)	0.008*
**Women (n = 95,240)**						
Lipophilic statin prescription	1.04 (1.01-1.07)	0.012*	0.94 (0.91-0.97)	<0.001*	0.94 (0.91-0.97)	<0.001*
**Low income (n = 95,240)**						
Lipophilic statin prescription	1.06 (1.02-1.10)	<0.001*	0.95 (0.92-0.99)	0.009*	0.95 (0.92-0.99)	0.014*
**High income (n = 99,545)**						
Lipophilic statin prescription	1.03 (1.00-1.06)	0.031*	0.93 (0.90-0.96)	<0.001*	0.93 (0.91-0.96)	<0.001*
**Urban residents (n = 76,535)**						
Lipophilic statin prescription	1.06 (1.02-1.10)	<0.001*	0.95 (0.92-0.99)	0.009*	0.95 (0.92-0.99)	0.014*
**Rural residents (n = 118,250)**						
Lipophilic statin prescription	1.03 (1.00-1.06)	0.031*	0.93 (0.90-0.96)	<0.001*	0.93 (0.91-0.96)	<0.001*
**Underweight (n = 4,857)**						
Lipophilic statin prescription	1.10 (0.91-1.34)	0.320	0.91 (0.74-1.11)	0.355	0.91 (0.74-1.11)	0.350
**Normal weight (n = 69,686)**						
Lipophilic statin prescription	1.05 (1.01-1.10)	0.02*	0.92 (0.88-0.97)	<0.001*	0.92 (0.88-0.97)	<0.001*
**Overweight (n = 52,331)**						
Lipophilic statin prescription	1.06 (1.02-1.11)	0.005*	0.95 (0.91-1.00)	0.034*	0.95 (0.91-1.00)	0.043*
**Obese (n = 67,911)**						
Lipophilic statin prescription	1.02 (0.99-1.06)	0.210	0.95 (0.91-0.98)	0.004*	0.95 (0.92-0.99)	0.006*
**Nonsmoker (n = 152,781)**						
Lipophilic statin prescription	1.02 (1.00-1.05)	0.055	0.93 (0.91-0.96)	<0.001*	0.93 (0.91-0.96)	<0.001*
**Past smoker and current smoker (n = 42,004)**						
Lipophilic statin prescription	1.11 (1.06-1.17)	<0.001*	0.97 (0.93-1.03)	0.326	0.97 (0.92-1.03)	0.305
**Alcohol consumption < 1 time a week (n = 155,947)**						

Lipophilic statin prescription	1.03 (1.00-1.05)	0.029*	0.93 (0.91-0.95)	<0.001*	0.93 (0.91-0.96)	<0.001*
**Alcohol consumption ≥ 1 time a week (n = 38,838)**						
Lipophilic statin prescription	1.11 (1.05-1.17)	<0.001*	0.99 (0.93-1.04)	0.666	0.99 (0.94-1.05)	0.719
**Systolic blood pressure < 120 mmHg and diastolic blood pressure < 80 mmHg (n = 55,815)**						
**Lipophilic statin prescription**	1.10 (1.05-1.15)	<0.001*	0.95 (0.91-1.00)	0.052	0.95 (0.90-1.00)	0.043*
**Systolic blood pressure ≥ 120 mmHg or diastolic blood pressure ≥ 80 mmHg (n = 138,970)**						
Lipophilic statin prescription	1.03 (1.00-1.06)	0.023*	0.94 (0.91-0.96)	<0.001*	0.94 (0.91-0.97)	<0.001*
**Fasting blood glucose < 100 mg/dL (n = 122,997)**						
Lipophilic statin prescription	1.04 (1.01-1.07)	0.019*	0.90 (0.87-0.93)	<0.001*	0.90 (0.87-0.93)	<0.001*
**Fasting blood glucose ≥ 100 mg/dL (n = 71,788)**						
Lipophilic statin prescription	1.08 (1.04-1.11)	<0.001*	0.98 (0.95-1.02)	0.381	0.99 (0.95-1.02)	0.436
**Total cholesterol < 200 mg/dL (n = 102,102)**						
Lipophilic statin prescription	1.04 (1.01-1.07)	0.007*	0.93 (0.90-0.96)	<0.001*	0.93 (0.91-0.96)	<0.001*
**Total cholesterol ≥ 200 mg/dL (n = 92,683)**						
Lipophilic statin prescription	1.05 (1.01-1.09)	0.011*	0.96 (0.92-1.00)	0.072	0.96 (0.92-1.00)	0.074
**Hemoglobin ≥ 13 g/dL for men and ≥ 12 g/dL for women (n = 168,718)**						
Lipophilic statin prescription	1.03 (1.00-1.05)	0.02*	0.93 (0.90-0.95)	<0.001*	0.93 (0.91-0.96)	<0.001*
**Hemoglobin < 13 g/dL for men and < 12 g/dL for women (n = 26,067)**						
Lipophilic statin prescription	1.12 (1.06-1.19)	<0.001*	1.01 (0.95-1.08)	0.688	1.01 (0.95-1.08)	0.683
**CCI scores = 0 (n = 117,143)**						
Lipophilic statin prescription	1.04 (1.01-1.08)	0.011*	0.93 (0.89-0.96)	<0.001*	0.93 (0.90-0.96)	<0.001*
**CCI scores = 1 (n = 32,408)**						
Lipophilic statin prescription	0.96 (0.91-1.01)	0.091	0.92 (0.88-0.97)	0.002*	0.92 (0.88-0.97)	<0.001*
**CCI scores ≥ 2 (n = 45,234)**						
Lipophilic statin prescription	1.04 (1.00-1.09)	0.037*	0.98 (0.94-1.02)	0.290	0.98 (0.94-1.02)	<0.001*
**No dyslipidemia history (n = 85,094)**						
Lipophilic statin prescription	0.94 (0.86-1.03)	0.205	0.94 (0.86-1.03)	0.156	0.93 (0.85-1.02)	0.129
**Dyslipidemia history (n = 109,691)**						
Lipophilic statin prescription	0.93 (0.91-0.96)	<0.001*	0.96 (0.93-0.98)	<0.001*	0.96 (0.94-0.98)	0.002*

Abbreviation: CCI, Charlson Comorbidity Index.* Conditional or unconditional logistic regression model, Significance at P < 0.05.† Models were stratified by age, sex, income, and region of residence. ‡ Model 1 was adjusted for systolic blood pressure, diastolic blood pressure, fasting blood glucose, total cholesterol, and hemoglobin levels.

Table S3 Subgroup analyses of crude and adjusted odds ratios (95% confidence interval) of hydrophilic statin prescription (per 1 year) for overall migraines

Characteristics	Odds ratios (95% CI)					
	Crude†	P-value	Model 1†‡	P-value	Model 2†§	P-value
**Age < 65 years old (n = 114,650)**						
Hydrophilic statin prescription	1.05 (0.98-1.12)	0.160	0.93 (0.87-1.00)	0.055	0.94 (0.88-1.01)	0.071
**Age ≥ 65 years old (n = 80,135)**						
Hydrophilic statin prescription	0.99 (0.94-1.04)	0.727	0.92 (0.88-0.97)	0.003*	0.93 (0.88-0.98)	0.005*
**Men (n = 67,470)**						
Hydrophilic statin prescription	1.03 (0.96-1.10)	0.450	0.92 (0.86-0.99)	0.019*	0.93 (0.86-0.99)	0.027*
**Women (n = 95,240)**						
Hydrophilic statin prescription	1.00 (0.95-1.06)	0.902	0.92 (0.87-0.97)	0.001*	0.92 (0.87-0.97)	0.002*
**Low income (n = 95,240)**						
Hydrophilic statin prescription	1.04 (0.98-1.10)	0.243	0.93 (0.88-0.99)	0.032*	0.94 (0.88-1.00)	0.039*
**High income (n = 99,545)**						
Hydrophilic statin prescription	0.99 (0.94-1.05)	0.776	0.91 (0.86-0.96)	<0.001*	0.91 (0.86-0.96)	0.001*
**Urban residents (n = 76,535)**						
Hydrophilic statin prescription	1.00 (0.94-1.07)	0.967	0.90 (0.85-0.96)	0.002*	0.91 (0.85-0.97)	0.003*
**Rural residents (n = 118,250)**						
Hydrophilic statin prescription	1.02 (0.97-1.08)	0.483	0.93 (0.88-0.98)	0.01*	0.93 (0.88-0.99)	0.013*
**Underweight (n = 4,857)**						
Hydrophilic statin prescription	0.89 (0.58-1.35)	0.585	0.71 (0.46-1.10)	0.126	0.71 (0.46-1.10)	0.128
**Normal weight (n = 69,686)**						
Hydrophilic statin prescription	1.05 (0.97-1.13)	0.234	0.93 (0.85-1.00)	0.062	0.93 (0.86-1.01)	0.069
**Overweight (n = 52,331)**						
Hydrophilic statin prescription	0.97 (0.89-1.05)	0.424	0.86 (0.80-0.94)	<0.001*	0.87 (0.80-0.94)	<0.001*
**Obese (n = 67,911)**						
Hydrophilic statin prescription	1.02 (0.96-1.08)	0.577	0.95 (0.90-1.01)	0.121	0.96 (0.90-1.02)	0.153
**Nonsmoker (n = 152,781)**						
Hydrophilic statin prescription	1.00 (0.95-1.05)	0.976	0.92 (0.87-0.96)	<0.001*	0.92 (0.88-0.96)	<0.001*
**Past smoker and current smoker (n = 42,004)**						
Hydrophilic statin prescription	1.06 (0.97-1.15)	0.206	0.92 (0.85-1.01)	0.068	0.92 (0.85-1.01)	0.068
**Alcohol consumption < 1 time a week (n = 155,947)**						

Hydrophilic statin prescription	0.99 (0.95-1.04)	0.726	0.90 (0.86-0.95)	<0.001*	0.91 (0.86-0.95)	<0.001*
**Alcohol consumption ≥ 1 time a week (n = 38,838)**						
Hydrophilic statin prescription	1.10 (1.00-1.20)	0.048*	0.98 (0.89-1.07)	0.626	0.98 (0.89-1.08)	0.696
**Systolic blood pressure < 120 mmHg and diastolic blood pressure < 80 mmHg (n = 55,815)**						
**Hydrophilic statin prescription**	1.02 (0.94-1.11)	0.599	0.89 (0.81-0.96)	0.005*	0.89 (0.82-0.97)	0.006*
**Systolic blood pressure ≥ 120 mmHg or diastolic blood pressure ≥ 80 mmHg (n = 138,970)**						
Hydrophilic statin prescription	1.01 (0.96-1.06)	0.657	0.93 (0.88-0.97)	0.002*	0.93 (0.89-0.98)	0.003*
**Fasting blood glucose < 100 mg/dL (n = 122,997)**						
Hydrophilic statin prescription	1.07 (1.01-1.14)	0.015*	0.94 (0.89-1.00)	0.056	0.95 (0.89-1.01)	0.076
**Fasting blood glucose ≥ 100 mg/dL (n = 71,788)**						
Hydrophilic statin prescription	0.98 (0.93-1.04)	0.566	0.90 (0.85-0.95)	<0.001*	0.90 (0.85-0.95)	<0.001*
**Total cholesterol < 200 mg/dL (n = 102,102)**						
Hydrophilic statin prescription	1.01 (0.96-1.06)	0.782	0.91 (0.87-0.96)	<0.001*	0.92 (0.87-0.97)	<0.001*
**Total cholesterol ≥ 200 mg/dL (n = 92,683)**						
Hydrophilic statin prescription	1.02 (0.95-1.10)	0.577	0.93 (0.87-1.01)	0.083	0.94 (0.87-1.01)	0.092
**Hemoglobin ≥ 13 g/dL for men and ≥ 12 g/dL for women (n = 168,718)**						
Hydrophilic statin prescription	1.01 (0.97-1.06)	0.596	0.92 (0.88-0.96)	<0.001*	0.92 (0.88-0.97)	<0.001*
**Hemoglobin < 13 g/dL for men and < 12 g/dL for women (n = 26,067)**						
Hydrophilic statin prescription	1.01 (0.91-1.12)	0.882	0.91 (0.81-1.01)	0.078	0.91 (0.81-1.01)	0.080
**CCI scores = 0 (n = 117,143)**						
Hydrophilic statin prescription	1.05 (0.99-1.11)	0.107	0.93 (0.88-0.99)	0.027*	0.94 (0.88-1.00)	0.04*
**CCI scores = 1 (n = 32,408)**						
Hydrophilic statin prescription	0.95 (0.87-1.03)	0.226	0.93 (0.85-1.01)	0.090	0.93 (0.85-1.01)	<0.001*
**CCI scores ≥ 2 (n = 45,234)**						
Hydrophilic statin prescription	0.95 (0.88-1.03)	0.192	0.90 (0.83-0.97)	0.005*	0.89 (0.83-0.96)	<0.001*
**No dyslipidemia history (n = 85,094)**						
Hydrophilic statin prescription	1.06 (0.90-1.23)	0.492	1.03 (0.88-1.20)	0.718	1.02 (0.88-1.19)	0.779
**Dyslipidemia history (n = 109,691)**						
Hydrophilic statin prescription	0.91 (0.87-0.95)	<0.001*	0.92 (0.89-0.97)	<0.001*	0.93 (0.89-0.97)	<0.001*

Abbreviation: CCI, Charlson Comorbidity Index.* Conditional or unconditional logistic regression model, Significance at $P < 0.05$.† Models were stratified by age, sex, income, and region of residence. ‡ Model 1 was adjusted for systolic blood pressure, diastolic blood pressure, fasting blood glucose, total cholesterol, and hemoglobin levels.
